# Supplementary material for: Developmental Changes of Immunity and Different Responses to Weaning Stress of Chinese Indigenous Piglets and Duroc Piglets during Suckling and Weaning Periods
Source: Int J Mol Sci. 2022 Dec 12;23(24):15781. doi: 10.3390/ijms232415781 (PMC9779383; doi:10.3390/ijms232415781)
Supplement: Supplementary file 1 [file ijms-23-15781-s001.zip › ijms-2069178-supplementary.pdf]

Supplementary Table S1. Primers sequences used for the RT-PCR.

Gene names	5′-3′ Primer sequence	Product size (bp)
CD14	F: CGTTTGTGGAGCCTGGAAG<br>R: TGCGGATGCGTGAAGTTG	125
IL-1β	F: AAGAGGGACATGGAGAAGCGATTTG<br>R: TTGTTCTGCTTGAGAGGTGCTGATG	114
IL-2	F: AAGCTCTGGAGGGAGTGCTA<br>R: CAACAGCAGTTACTGTCTCATCA	115
IL-6	F: GCTGCTTCTGGTGATGGCTACTG<br>R: AGAGGTGAAGAGCATTTTGTCTGAGG	97
IL-10	F: GTCCGACTCAACGAAGAAGG<br>R: GCCAGGAAGATCAGGCAATA	106
IL-17	F: GCACACGGGCTGCATCAACG<br>R: TGCAACCAACAGTGACCCGCA	124
IRAK1	F: CAAGGCAGGTCAGGTTTCGT<br>R: TTCGTGGGGCGTGTAGTGT	126
IFN-γ	F: CCATTCAAAGGAGCATGGAT<br>R: GAGTTCACTGATGGCTTTGC	146
LBP	F: GAACACAGCCGAATGGTCTAC<br>R: GGAAGGAGTTGGTGGTCAGT	126
MD2	F: TGCAATTCCTCTGATGCAAG<br>R: CCACCATATTCTCGGCAAAT	162
MyD88	F: GATGGTAGCGGTTGTCTCTGAT<br>R: GATGCTGGGGAACTCTTTCTTC	146
NF-κB	F: AGTACCCTGAGGCTATAACTCGC<br>R: TCCGCAATGGAGGAGAAGTC	109
NOD1	F: CTGTCGTCAACACCGATCCA<br>R: CCAGTTGGTGACGCAGCTT	134
NOD2	F: GAGCGCATCCTCTTAACTTTCG<br>R: ACGCTCGTGATCCGTGAAC	105
RIP2	F: CAGTGTCCAGTAAATCGCAGTTG<br>R: CAGGCTTCCGTCATCTGGTT	159
TNF-α	F: ATTCAGGGATGTGTGGCCTG<br>R: CCAGATGTCCCAGGTTGCAT	141
TRAF6	F: CAAGAGAATACCCAGTCGCACA<br>R: ATCCGAGACAAAGGGGAAGAA	114
TLR-2	F: GCAATAATGACACCTTCGCTGAGATTC<br>R: AGATGGCTGATGTTCTGAATTGACCTC	139
TLR-4	F: AGGACGAAGACTGGGTGAGGAATG<br>R: CCTGGATGATGTTAGCAGCGATGG	126
β-actin	F: GATCTGGCACCACACCTTCTACAAC<br>R: TCATCTTCTCACGGTTGGCTTTGG	107

IL, interleukin**;** *TNF*, tumor necrosis factor; *IRAK1*, interleukin-1 receptor-associated kinase 1; *LBP*, lipopolysaccharide binding protein; *TRAF6*, tumor necrosis factor receptor-associated factor 6; *MD-2*, myeloid differential protein-2; *NOD*, nucleotide binding oligomerization domain; *CD14*, cluster of differentiation 14; *MyD88*, myeloid differentiation factor 88; *NF-κB*, nuclear factor kappa B; *RIP2*, receptor-interact protein 2.

Supplementary Table S2. Changes in blood cell-related hematological parameters in different pig breeds during suckling and weaning periods.

Items	TB	XB	DR	SEM	*p* Values
Red blood cell count (RBC, 10^{12}/L)					
1 day of age	5.44^{C}	5.51^{C}	5.64^{C}	0.14	0.834
10 days of age	5.28^{Cb}	5.93^{BCb}	6.80^{Ba}	0.18	0.001
21 days of age	6.50^{Bb}	6.52^{Bb}	7.83^{Aa}	0.16	<0.001
24 days of age	7.97^{A}	7.82^{A}	8.43^{A}	0.13	0.124
SEM	0.20	0.17	0.21		
p values	<0.001	<0.001	<0.001		
Hemoglobin (HGB, g/L)					
1 day of age	97.50^{C}	95.80^{C}	103.30^{C}	2.64	0.493
10 days of age	99.60^{Cc}	112.10^{Bb}	127.60^{Ba}	2.64	<0.001
21 days of age	126.40^{Bab}	119.90^{Bb}	134.60^{ABa}	2.13	0.013
24 days of age	141.60^{A}	139.20^{A}	142.60^{A}	1.84	0.755
SEM	3.42	2.88	3.06		
p values	<0.001	<0.001	<0.001		
Hematocrit (HCT, %)					
1 day of age	31.18^{C}	31.84^{C}	33.23^{B}	0.86	0.626
10 days of age	33.39^{Cc}	38.52^{Bb}	41.41^{Aa}	0.81	<0.001
21 days of age	39.13^{Bb}	39.07^{Bb}	44.01^{Aa}	0.62	<0.001
24 days of age	44.77^{A}	45.30^{A}	43.72^{A}	0.58	0.539
SEM	1.00	0.88	0.92		
p values	<0.001	<0.001	<0.001		
Mean corpuscular volume (MCV, fL)					
1 day of age	57.31^{B}	57.74^{B}	59.23^{AB}	0.81	0.613
10 days of age	63.67^{A}	65.73^{A}	61.08^{A}	0.97	0.145
21 days of age	60.67^{ABa}	60.08^{Ba}	56.37^{Bb}	0.79	0.048
24 days of age	56.33^{Ba}	58.04^{Ba}	52.01^{Cb}	0.73	0.001
SEM	0.83	0.80	0.83		
p values	0.004	<0.001	<0.001		
Mean corpuscular hemoglobin (MCH, pg)					
1 day of age	17.89^{B}	17.40^{C}	18.36^{A}	0.20	0.149
10 days of age	19.03^{AB}	19.06^{A}	18.81^{A}	0.25	0.911
21 days of age	19.56^{Aa}	18.41^{ABb}	17.31^{Bc}	0.25	<0.001
24 days of age	17.83^{Ba}	17.86^{BCa}	16.95^{Bb}	0.18	0.062
SEM	0.23	0.19	0.20		
p values	0.010	0.007	0.001		
Mean corpuscular hemoglobin concentration (MCHC, g/L)					
1 day of age	312.60^{Aa}	301.40^{Ab}	311.20^{Ba}	2.05	0.046
10 days of age	299.20^{Bab}	290.40^{Bb}	307.90^{Ba}	2.49	0.011
21 days of age	322.80^{Aa}	306.80^{Ab}	307.33^{Bb}	2.58	0.011
24 days of age	316.30^{Ab}	307.80^{Ac}	326.30^{Aa}	2.00	<0.001
SEM	2.13	1.88	2.17		
P Values	<0.001	0.001	0.003		
Coefficient variation of red blood cell volume distribution width (RDW-CV, %)					
1 day of age	19.55^{Cb}	20.31^{Cb}	27.79^{Aa}	0.95	<0.001
10 days of age	27.65^{A}	26.83^{A}	25.81^{AB}	0.54	0.387

21 days of age	24.57^{B}	22.92^{B}	22.42^{B}	0.45	0.122
24 days of age	21.76^{C}	22.35^{B}	22.70^{B}	0.37	0.601
SEM	0.61	0.46	0.69		
p values	<0.001	<0.001	0.011		
Standard deviation in red cell distribution width (RDW-SD, fL)					
1 day of age	43.18^{Cb}	45.12^{Cb}	64.31^{Aa}	2.85	0.001
10 days of age	67.38^{A}	66.37^{A}	60.81^{A}	1.90	0.329
21 days of age	58.73^{Ba}	53.42^{Bab}	49.27^{Bb}	1.55	0.038
24 days of age	47.85^{C}	49.98^{BC}	45.80^{B}	0.89	0.160
SEM	1.98	1.61	2.23		
p values	<0.001	<0.001	0.004		
Platelet (PLT, 10^{9}/L)					
1 day of age	376.30^{B}	381.70^{C}	418.00^{B}	17.33	0.581
10 days of age	769.20^{Aa}	860.20^{Aa}	563.10^{Ab}	40.90	0.006
21 days of age	625.00^{A}	585.90^{B}	531.44^{A}	27.18	0.383
24 days of age	610.50^{A}	690.80^{B}	562.00^{A}	31.44	0.247
SEM	36.85	35.25	21.14		
p values	0.001	<0.001	0.041		
Mean platelet volume (MPV, fL)					
1 day of age	10.57^{A}	10.15^{A}	10.65^{A}	0.13	0.222
10 days of age	10.24^{Aa}	9.59^{Bb}	10.26^{Aa}	0.12	0.035
21 days of age	9.24^{Bb}	9.70^{ABab}	10.10^{Aa}	0.12	0.010
24 days of age	9.46^{B}	8.95^{C}	9.33^{B}	0.12	0.179
SEM	0.13	0.11	0.13		
p values	<0.001	<0.001	0.001		
Platelet distribution width (PDW)					
1 day of age	15.85^{Ab}	16.28^{Aa}	16.25^{Aa}	0.06	0.002
10 days of age	15.87^{Aa}	15.89^{Ba}	15.47^{Bb}	0.05	<0.001
21 days of age	15.33^{B}	15.50^{C}	15.32^{BC}	0.05	0.241
24 days of age	15.17^{Bb}	15.52^{Ca}	15.12^{Cb}	0.07	0.041
SEM	0.06	0.06	0.09		
p values	<0.001	<0.001	<0.001		
Plateletcrit (PCT, %)					
1 day of age	0.40^{B}	0.39^{C}	0.44^{B}	0.02	0.410
10 days of age	0.69^{Aa}	0.76^{Aa}	0.58^{Ab}	0.02	0.002
21 days of age	0.57^{A}	0.57^{B}	0.54^{AB}	0.02	0.817
24 days of age	0.58^{A}	0.62^{B}	0.52^{AB}	0.03	0.362
SEM	0.02	0.03	0.02		
p values	0.001	<0.001	0.074		

Data are presented as means with their pooled SEM (n = 10). Different uppercase letters in the same column indicate significant differences among different days of age ($P < 0.05$), and different lowercase letters in the same row indicate significant differences among different pig breeds ($P < 0.05$). DR, Duroc piglet; TB, Taoyuan Black piglet; XB, Xiangcun Black piglet.
